# Altered brain functional network connectivity and topology in type 2 diabetes mellitus

**DOI:** 10.3389/fnins.2025.1472010

**Published:** 2025-01-28

**Authors:** Weiwei Ni, Weiyin Vivian Liu, Mingrui Li, Shouchao Wei, Xuanzi Xu, Shutong Huang, Lanhui Zhu, Jieru Wang, Fengling Wen, Hailing Zhou

**Affiliations:** ^1^Physical Examination Centre, Central People's Hospital of Zhanjiang, Zhanjiang, China; ^2^MR Research, GE Healthcare, Beijing, China; ^3^Department of Magnetic Resonance Imaging, Zhanjiang First Hospital of Traditional Chinese Medicine, Zhanjiang, China; ^4^Central People's Hospital of Zhanjiang, Zhanjiang Institute of Clinical Medicine, Zhanjiang, China; ^5^Department of Teaching and Training, Central People's Hospital of Zhanjiang, Zhanjiang, China; ^6^Department of Clinical Laboratory, Central People's Hospital of Zhanjiang, Zhanjiang, China; ^7^Department of Radiology, Central People's Hospital of Zhanjiang, Zhanjiang, China

**Keywords:** type 2 diabetes mellitus, functional magnetic resonance imaging, blood oxygenation level-dependent imaging, brain functional network connectivity, graph theory

## Abstract

**Introduction:**

Type 2 diabetes mellitus (T2DM) accelerates brain aging and disrupts brain functional network connectivity, though the specific mechanisms remain unclear. This study aimed to investigate T2DM-driven alterations in brain functional network connectivity and topology.

**Methods:**

Eighty-five T2DM patients and 67 healthy controls (HCs) were included. All participants underwent clinical, neuropsychological, and laboratory tests, followed by MRI examinations, including resting-state functional magnetic resonance imaging (rs-fMRI) and three-dimensional high-resolution T1-weighted imaging (3D-T1WI) on a 3.0 T MRI scanner. Post-image preprocessing, brain functional networks were constructed using the Dosenbach atlas and analyzed with the DPABI-NET toolkit through graph theory.

**Results:**

In T2DM patients, functional connectivity within and between the default mode network (DMN), frontal parietal network (FPN), subcortical network (SCN), ventral attention network (VAN), somatosensory network (SMN), and visual network (VN) was significantly reduced compared to HCs. Conversely, two functional connections within the VN and between the DMN and SMN were significantly increased. Global network topology analysis showed an increased shortest path length and decreased clustering coefficient, global efficiency, and local efficiency in the T2DM group. MoCA scores were negatively correlated with the shortest path length and positively correlated with global and local efficiency in the T2DM group. Node network topology analysis indicated reduced clustering coefficient, degree centrality, eigenvector centrality, and nodal efficiency in multiple nodes in the T2DM group. MoCA scores positively correlated with clustering coefficient and nodal efficiency in the bilateral precentral gyrus in the T2DM group.

**Discussion:**

This study demonstrated significant abnormalities in connectivity and topology of large-scale brain functional networks in T2DM patients. These findings suggest that brain functional network connectivity and topology could serve as imaging biomarkers, providing insights into the underlying neuropathological processes associated with T2DM-related cognitive impairment.

## 1 Introduction

Type 2 diabetes mellitus (T2DM) affects approximately 450 million adults worldwide (American Diabetes Association Professional Practice, 2024), leading to systemic complications. This chronic metabolic disease accelerates brain aging (Biessels et al., 2020) and nerve damage, resulting in cognitive impairment. T2DM facilitates the progression from mild cognitive impairment (MCI) to dementia, significantly diminishing quality of life. Understanding the mechanisms underlying cognitive impairment in T2DM is crucial for early detection and treatment (Srikanth et al., 2020).

Neurodegenerative and neuropsychological disorders, including T2DM, often have a prolonged preclinical phase lasting a decade or more. Subtle changes in brain function frequently precede overt structural neuropathology and behavioral symptoms. T2DM is characterized by hyperinsulinemia as reduced insulin receptor expression and receptor-activating enzymes, leading to the deposition of amyloid beta (Aβ) and Tau proteins (Arnold et al., 2018). Chronic hyperglycemia and insulin resistance disrupt neuronal function, synaptic plasticity and neurovascular integrity, contributing to neuroinflammation and oxidative stress exacerbate damage (van Sloten et al., 2020). The brain volume reduction and cognitive decline in associated brain regions (Zhang et al., 2014; Cao et al., 2024). Advancements in neuroimaging, particularly resting-state functional magnetic resonance imaging (rs-fMRI), have enabled the identification of numerous neuroimaging biomarkers. This non-invasive, repeatable, and straightforward technique has revealed alterations in blood oxygenation level-dependent (BOLD) signals in T2DM patients (Marquez and Yassa, 2019; Van Bussel et al., 2017; Zackova et al., 2021; Franke and Gaser, 2019). Post-processing of BOLD images facilitates the classification and mapping of brain functional networks, offering new insights into the pathophysiological mechanisms of T2DM-related cognitive disorders (Wang et al., 2016; Lv et al., 2018; Cole et al., 2010).

Significant progress has been made in understanding T2DM-driven brain functional networks. Both reduced and increased functional connectivity between various brain regions in T2DM patients have been observed (Cui et al., 2016; Liu et al., 2018; Li et al., 2020; Liu et al., 2017; Tan et al., 2019). Earlier studies primarily focused on local brain region connections, but it is now recognized that most cognitive functions involve multiple brain regions working together. Consequently, initial changes in brain regions were often overlooked (Fuster, 2006). The focus has shifted toward systematically identifying and understanding the functional organization of large-scale brain networks and their roles in cognitive and emotional processing (Bressler and Menon, 2010). Few studies, however, have examined changes in T2DM-related large-scale networks, with the default mode network (DMN) receiving more attention (Chen et al., 2015; Liu et al., 2019; Chen et al., 2016; Deng et al., 2021; Cui et al., 2015) while other large-scale networks are not fully understood. At present, the mechanism between changes in large-scale network functional connectivity and cognitive dysfunction is still not clearly understood. Graph theory analysis can elucidate important topological features of the whole brain, including small-world attributes, modulators, and hub nodes (Farahani et al., 2019). Several studies have reported differences in graph theory-derived parameters of brain functional networks between T2DM patients and healthy individuals. However, these studies often included small sample sizes and did not simultaneously analyze large-scale brain network connectivity and topological changes in T2DM patients (van Bussel et al., 2016; Xin et al., 2024; Zhou et al., 2022; Zhang et al., 2021). Differences in graph theory-derived parameters of brain functional networks between T2DM patients and healthy individuals are reported based on small cohorts without analyzing large-scale brain network connectivity and topological changes.

This study aimed to construct large-scale network connections in T2DM patients using BOLD-based rs-fMRI data and to display whole-brain and nodal topological features through graph theory analysis. This approach seeks to enhance the understanding of the mechanisms underlying cognitive dysfunction in T2DM and provide further ideas for the delay or prevention of brain disease in T2DM patients.

## 2 Materials and methods

### 2.1 Participants

This study was approved by the ethics committee of our hospital. Participants, all right-handed Han Chinese native speakers, attended the endocrinology department and health examination center between November 2022 and January 2024. Written informed consent was secured from all participants prior to enrollment. Inclusion criteria for T2DM group: 1. diagnoses of type 2 diabetes were established by an endocrinologist following the American Diabetes Association guidelines, and inclusion criteria for T2DM group were: 1. hyperglycemia was diagnosed with typical diabetic symptoms plus glycated hemoglobin A1C (HbA1c) ≥6.5% or fasting blood glucose (FBG) ≥7.0 mmol/L or fasting two-hour blood glucose (OGTT2h) ≥11.1 mmol/L or random FBG ≥ 11.1 mmol/L. Fasting was defined as no energy intake for at least 8 h prior to the examination; OGTT uses 75 g of anhydrous glucose dissolved in 300 mL of warm water (American Diabetes Association Professional Practice, 2024); 2. aged 18–65, Han nationality, right-handed; 3. blood pressure is normal; 4. complete cognitive function assessment and MRI examination. The inclusion criteria for the healthy controls (HCs) group were as follows: 1. those with matched gender, age and years of education to the T2DM group; 2. no history of diabetes and normal blood pressure; 3. complete cognitive function assessment and MRI examination. Exclusion criteria included individuals younger than 18 or older than 65 years, those with organic central nervous system diseases, a history of mental illness or familial mental illness, severe head trauma, severe hypoglycemia, significant vascular complications, alcohol dependence or substance abuse, noticeable hearing or visual impairments, women who were pregnant, breastfeeding, or using contraceptives, and those with contraindications for MRI. In total, 85 T2DM patients and 67 HCs were enrolled. Demographic data, including gender, age, and education, were self-reported by all participants.

### 2.2 Laboratory analysis and cognition testing

Blood samples were obtained from all subjects to measure glycated hemoglobin A_1c_ (HbA_1c_), fasting blood glucose (FBG), triglyceride (TG), total cholesterol (TC), and low-density lipoprotein (LDL) levels. All participants underwent neuropsychological assessments, including the Montreal cognitive assessment (MoCA) (Li et al., 2018), the digital span test (DST, comprising forward and backward tasks) (Diamond, 2013), and the clock drawing test (CDT) (Zhou et al., 2010), with each test lasting 5–10 min.

### 2.3 MRI acquisition

All participants were subjected to MRI examinations on a 3.0 T MRI scanner (SIGNA Pioneer, GE Healthcare) with a 16-channel phased-array head coil. Routine imaging included T1-weighted, T2-weighted, and T2-fluid attenuated inversion recovery (T2-FLAIR) sequences to rule out organic brain lesions. Blood oxygenation level-dependent (BOLD) imaging was then conducted with the following parameters: flip angle = 90.0°, bandwidth = 250.0 kHz, TR = 2000 ms, TE = 30.0 ms, slices = 48, thickness = 3.0 mm, pixel size = 3.0 × 3.0 × 3.0 mm^3^, FOV = 256 × 256 mm^2^, and NEX = 1. Additionally, three-dimensional high-resolution T1-weighted brain volume imaging (3D-T1WI BRAVO) was performed with these parameters: flip angle = 12.0°, bandwidth = 31.25 kHz, TR = 7.8 ms, TE = 3.1 ms, slices = 1,024, thickness = 1.0 mm, pixel size = 1.0 × 1.0 × 1.0 mm^3^, FOV = 25.6 × 25.6 cm^2^, and NEX = 1.

### 2.4 Assessment of small-vessel disease

White matter degeneration and lacunar infarction in five brain regions (bilateral frontal lobes, parietal and occipital lobes, temporal lobes, cerebellum and brainstem, and basal ganglia) were quantitatively assessed on T2-FLAIR images using the age-related white matter changes (ARWMC) Wahlund score (Wahlund et al., 2001). These assessments were conducted by two experienced radiologists who were blinded to the group assignments. Discrepancies were resolved through joint discussion to reach consensus. Participants with ratings above 2 were excluded from the study.

### 2.5 Image processing

All rs-fMRI data were processed using SPM12 and DPABI (version 8.1) toolkits in MATLAB (Yan et al., 2016). The preprocessing steps were as follows: 1. DICOM-NIFTI data format conversion. 2. Removal of the first 10 time points. 3. Temporal correction. 4. Head movement correction: ensure that the head motion displacement of all enrolled subjects is <2 mm and the head motion rotation is <2 °, and the mean FD_Jenkinson is obtained as the covariate for the subsequent comparison between the groups. 5. Nuisnace covariate regression: to remove the nuisance signals, the Friston 24-parameter model was utilized to regress out head motion effects from the realigned data. The signals from WM and CSF were regressed out to reduce respiratory and cardiac effects. 6. Spatial normalization: We first registered the 3D-T1WI images to the fMRI images. We selected the “New Segment + Dartel” option. The “New Segment + Dartel” option performs the New Segment operation on the 3D-T1WI structural images to obtain tissue maps for gray matter, white matter, and cerebrospinal fluid. Additionally, DARTEL transforms the structural and tissue images into MNI space and generates transformation matrices. Finally, we chose the “Normalize by DARTEL” option, which uses the transformation matrices to convert the functional images into MNI space. 7. Filtering: select signals in the frequency band of 0.01 Hz to 0.08 Hz. These procedures were applied to enhance image data quality, reduce confounding factors, and prepare the data for further analysis.

### 2.6 Construction of brain function networks

A total of 142 ROIs from the Dosenbach atlas were used as nodes and classified into seven large-scale networks (Dosenbach et al., 2010; Yeo et al., 2011). Seven large-scale networks include: default mode network (DMN), frontal parietal network (FPN), subcortical network (SCN), ventral attention network (VAN), somatosensory network (SMN), visual network (VN), and dorsal attention network (DAN). The BOLD signals for each node were averaged, and Pearson correlation coefficients were calculated to determine the correlation between BOLD signals across nodes. Functional connectivity (FC) between nodes was then computed using Z transformation, with these connections represented as edges within the brain functional network. Since the current negative functional connection is still controversial, we set the negative functional connection to 0 and keep only the positive functional connection, so as to obtain the undirected weighted positive resting state functional connection matrix (Pang et al., 2022). The details, including node names, networks, and MNI coordinates, are listed in Supplementary Table S1.

### 2.7 Graph theory analysis of brain functional networks

The topological attributes of the brain functional network were computed across sparsity levels ranging from 0.1 to 0.34, with a step size of 0.01 (Yang et al., 2021), using the DPABI-NET toolkit in MATLAB. Global topological attributes assessed included sigma, gamma, lambda, shortest path length, clustering coefficient, global efficiency, and local efficiency. Node-specific attributes evaluated comprised clustering coefficient, degree centrality, eigenvector centrality, and nodal efficiency.

### 2.8 Statistics

Statistical analyses were performed using SPSS (IBM, SPSS, version 25). Differences in demographic data and neurocognitive test scores between groups were assessed using either independent two-sample t-tests or Mann–Whitney U tests, based on the distribution normality and variance equality of the data. Chi-square tests were used to evaluate gender differences between groups. Connectivity between functional network nodes was compared with two-sample *t-*tests, adjusting for gender, age, education level, and average head motion. Results were corrected for false discovery rate (FDR) using the Benjamini-Hochberg (B-H) procedure, with statistical significance set at *p* < 0.05.

Network topology attributes were analyzed using analysis of covariance (ANCOVA), incorporating gender, age, education level, and average head motion as covariates. The significance threshold for global network topology attributes was set at *p* < 0.05. For node-specific attributes, FDR correction was applied, with significance defined as *Q* < 0.05 after adjustment. Gender, age, education level and average head movement were used as covariables to conduct partial correlation analysis of MoCA scores, clinical measurements, and brain network topological parameters in the T2DM group, with statistical significance determined as *p* < 0.05.

## 3 Results

### 3.1 Demographic data, clinical measurements, and cognitive testing

Gender, age, and education were not comparable between the T2DM and HC groups. However, the T2DM group exhibited significantly lower MoCA scores than the HC group (Table 1).

**Table 1:** Demographic data, clinical biochemical indicators and neuropsychological result of all subjects.

	T2DM (*n* = 85)	HC (*n* = 67)	Statistics	*p*-value
Age (years)	50.29 ± 9.62	47.88 ± 8.05	*t* = 1.648	0.101
Sex (female/male)	30/55	31/36	*χ2* = 1.878	0.171
Education (years)	12.00 (9.00;15.00)	12 (9.00;16.00)	*z* = −0.087	0.930
HbA1c (%)	8.91 ± 2.06	N/A	N/A	N/A
FBG (mmol/L)	7.88 (6.77;10.63)	N/A	N/A	N/A
TG (mmol/L)	1.89 (1.11;2.52)	N/A	N/A	N/A
TC (mmol/L)	5.16 ± 1.27	N/A	N/A	N/A
LDL (mmol/L)	3.13 (2.58;3.84)	N/A	N/A	N/A
CDT	3.00 (3.00;4.00)	3.00 (3.00;4.00)	*z* = −0.355	0.722
MoCA	26.00 (24.00;27.00)	27.00 (27.00;29.00)	*z* = −5.831	<0.001*
DST(forward)	8.00 (7.00;9.00)	8.00 (8.00;9.00)	*z* = −1.105	0.269
DST(inverse)	4.00 (3.00;5.00)	4.00 (3.00;4.00)	*z* = −0.276	0.783

FBG, fasting blood glucose; TG, triglyceride; TC, total cholesterol; LDL, low-density lipoprotein; DST, the digital span test; CDT, the clock drawing test; MoCA, Montreal cognitive assessment. *P < 0.05.

### 3.2 Changes in connectivity of large-scale functional networks

**Figure 1:**
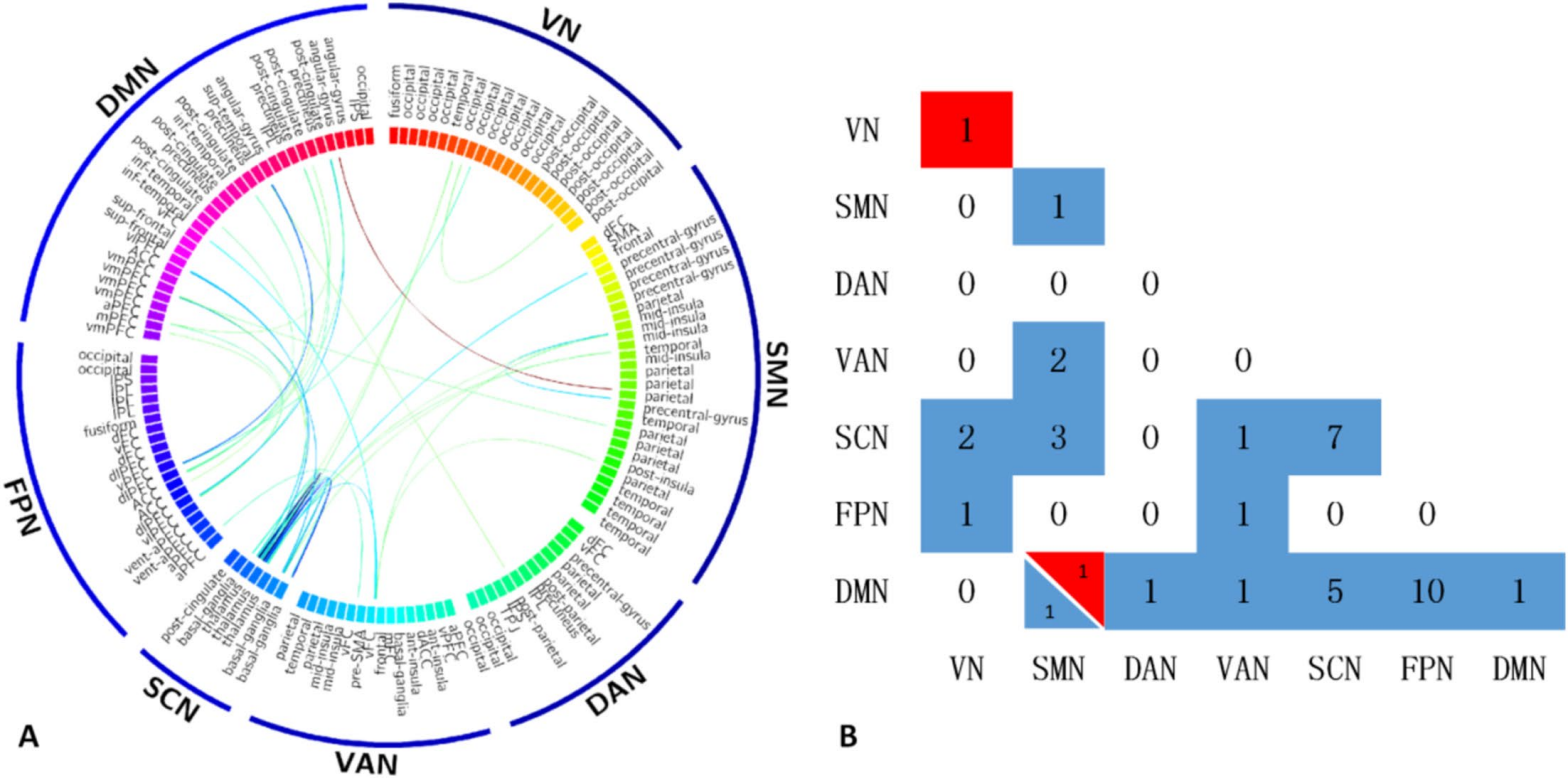
The T2DM group showed significantly reduced functional connections within and between the DMN, FPN, SCN, VAN, DAN, SMN, and VN compared to the HC group. However, two functional connections were significantly increased within the VN and between the DMN and SMN **(A)**. Maps show the number of significant edges for each pair of networks for each of the two contrasts **(B)**. DMN, default mode network; FPN, frontal parietal network; SCN, subcortical network; VAN, ventral attention network; SMN, somatosensory network; VN, visual network; DAN, dorsal attention network.

The T2DM group showed significantly reduced functional connections within and between the DMN, FPN, SCN, VAN, DAN, SMN, and VN compared to the HC group. However, two functional connections were significantly increased within the VN and between the DMN and SMN (Figure 1).

### 3.3 Group differences in global network metrics

**Figure 2:**
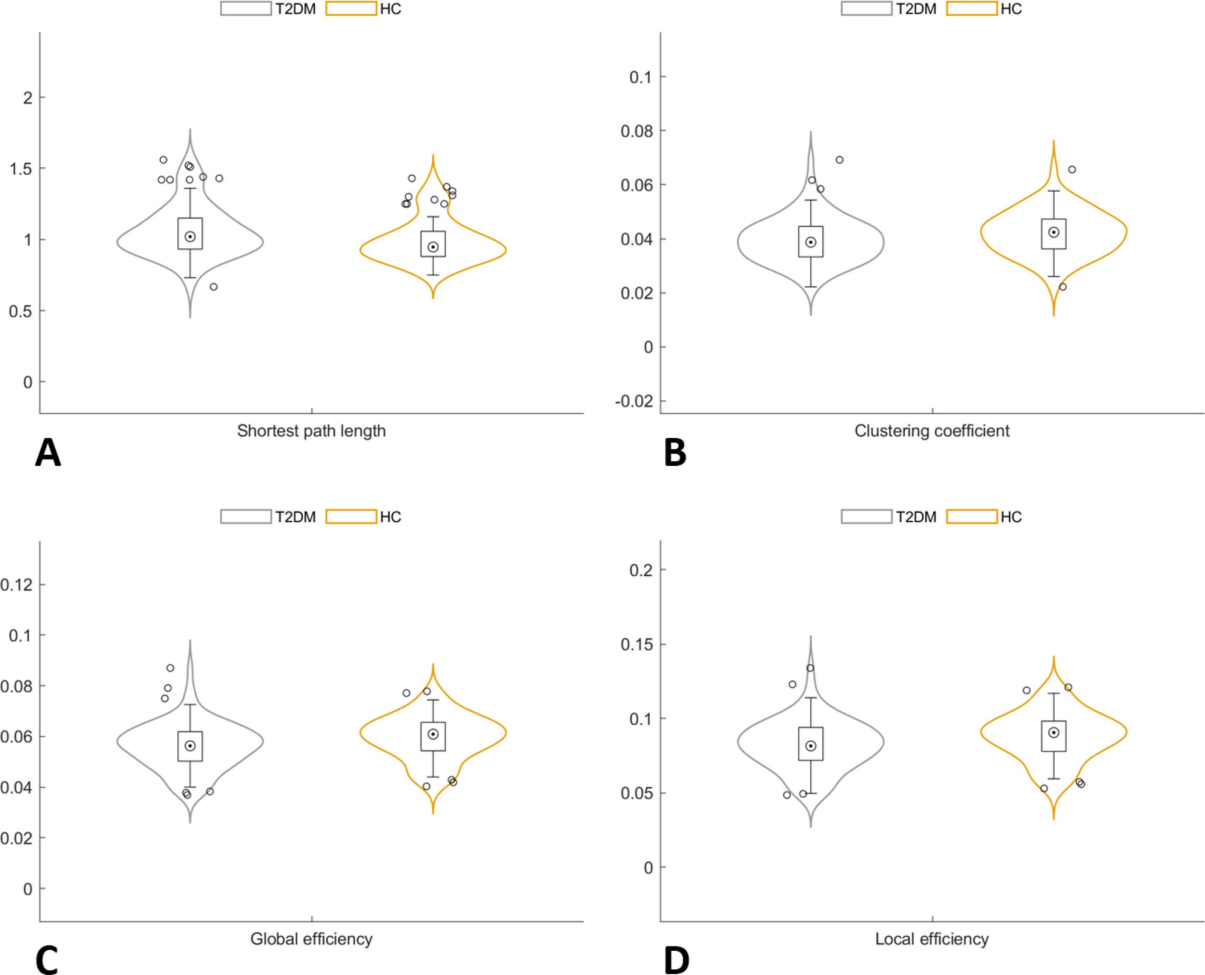
In the global network topology analysis, the T2DM group showed a higher shortest path length **(A)**, along with a lower clustering coefficient **(B)**, global efficiency **(C)** and local efficiency **(D)** compared to the HC group.

In the global network topology analysis, the T2DM group showed a lower clustering coefficient, global efficiency, and local efficiency, along with a higher shortest path length compared to the HC group. No significant differences were observed in sigma, gamma, and lambda between the groups (Table 2; Figure 2).

**Table 2:** Global network metrics of all subjects.

	T2DM (*n* = 85)	HC (*n* = 67)	Statistics(*F*)	*P-*value
Lp	1.059 ± 0.185	0.992 ± 0.158	5.991	0.016*
Cp	0.039 ± 0.009	0.042 ± 0.008	4.651	0.033*
Eg	0.056 ± 0.009	0.060 ± 0.009	6.131	0.014*
Eloc	0.082 ± 0.017	0.088 ± 0.016	5.858	0.017*
Sigma	1.195 ± 0.229	1.202 ± 0.229	0.021	0.884
Gamma	0.409 ± 0.066	0.417 ± 0.069	0.113	0.737
Lambda	0.089 ± 0.004	0.090 ± 0.004	1.428	0.234

Lp, shortest path length; Cp, clustering coefficient; Eg, global efficiency; Eloc, local efficiency. *P < 0.05.

### 3.4 Group differences in node-level network metrics

**Figure 3:**
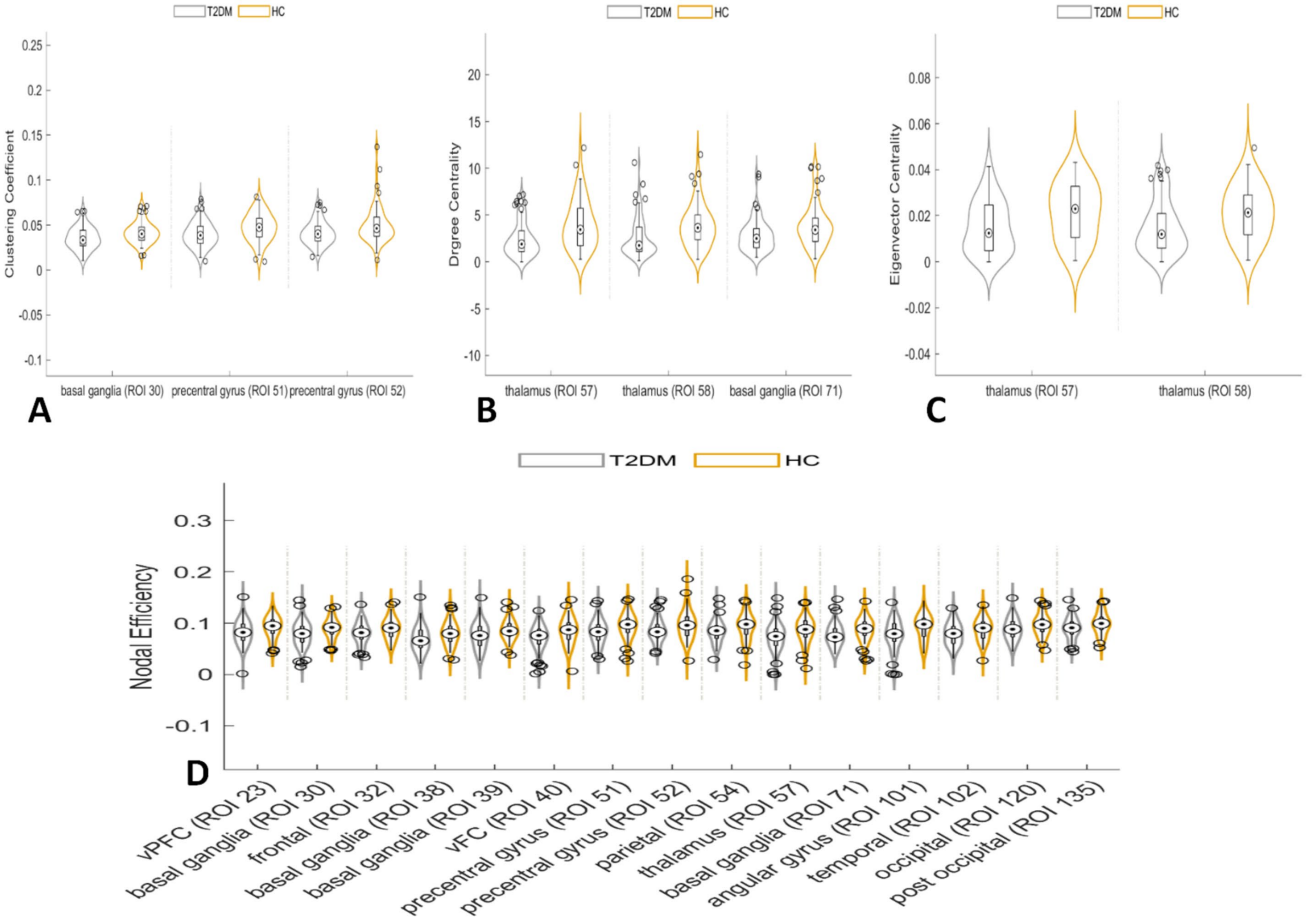
In the node network topology analysis, the clustering coefficient was lower in the left basal ganglia and bilateral precentral gyrus in the T2DM group compared to the HC group **(A)**. Degree centrality and eigenvector centrality were reduced in the bilateral thalamus, and degree centrality alone was decreased in the right basal ganglia **(B,C)**. Nodal efficiency was also lower in several brain regions **(D)**.

In the node network topology analysis, the clustering coefficient was lower in the left basal ganglia and bilateral precentral gyrus in the T2DM group compared to the HC group. Degree and eigenvector centrality were reduced in the bilateral thalamus, and degree centrality alone was decreased in the right basal ganglia. Nodal efficiency was also lower in several brain regions (Table 3; Figure 3).

**Table 3:** Node-level network metrics of all subjects.

Node name	MNI coordinates			Statistics(*F*)	*P*-value	*Q*-value
	X	Y	Z			
Clustering coefficient						
Basal ganglia (L) (ROI 30)	−6	17	34	12.729	0.000	0.035*
Precentral gyrus (R) (ROI 51)	46	−8	24	13.748	0.000	0.042*
Precentral gyrus (L) (ROI 52)	−54	−9	23	11.730	0.001	0.038*
Degree centrality						
Thalamus (L) (ROI 57)	−12	−12	6	21.270	0.000	0.001*
Thalamus (R) (ROI 58)	11	−12	6	15.468	0.000	0.009*
Basal ganglia (R) (ROI 71)	11	−24	2	11.579	0.001	0.041*
Eigenvector centrality						
Thalamus (L) (ROI 57)	−12	−12	6	14.025	0.000	0.037*
Thalamus (R) (ROI 58)	11	−12	6	13.172	0.000	0.028*
Nodal efficiency						
vPFC (L) (ROI 23)	−52	28	17	9.454	0.003	0.045*
Basal ganglia (L) (ROI 30)	−6	17	34	11.681	0.001	0.039*
Frontal (R) (ROI 32)	58	11	14	8.176	0.005	0.049*
Basal ganglia (L) (ROI 38)	−20	6	7	9.923	0.002	0.047*
Basal ganglia (R) (ROI 39)	14	6	7	8.120	0.005	0.047*
vFC (L) (ROI 40)	−48	6	1	12.170	0.001	0.046*
Precentral gyrus (R) (ROI 51)	46	−8	24	9.755	0.002	0.044*
Precentral gyrus (L) (ROI 52)	−54	−9	23	9.308	0.003	0.043*
Parietal (L) (ROI 54)	−47	−12	36	8.739	0.004	0.043*
Thalamus (L) (ROI 57)	−12	−12	6	11.574	0.001	0.031*
basal ganglia (R) (ROI 71)	11	−24	2	8.832	0.003	0.045*
Angular gyrus (L) (ROI 101)	−41	−47	29	10.189	0.002	0.049*
Temporal (L) (ROI 102)	−59	−47	11	9.122	0.003	0.042*
Occipital (R) (ROI 120)	19	−66	−1	8.658	0.004	0.041*
Post occipital (L) (ROI 135)	−5	−80	9	8.097	0.005	0.045*

vPFC, ventromedial prefrontal cortex; vFC, ventral frontal cortex; *Q < 0.05.

### 3.5 Correlation between network theory parameters and MoCA scores

**Figure 4:**
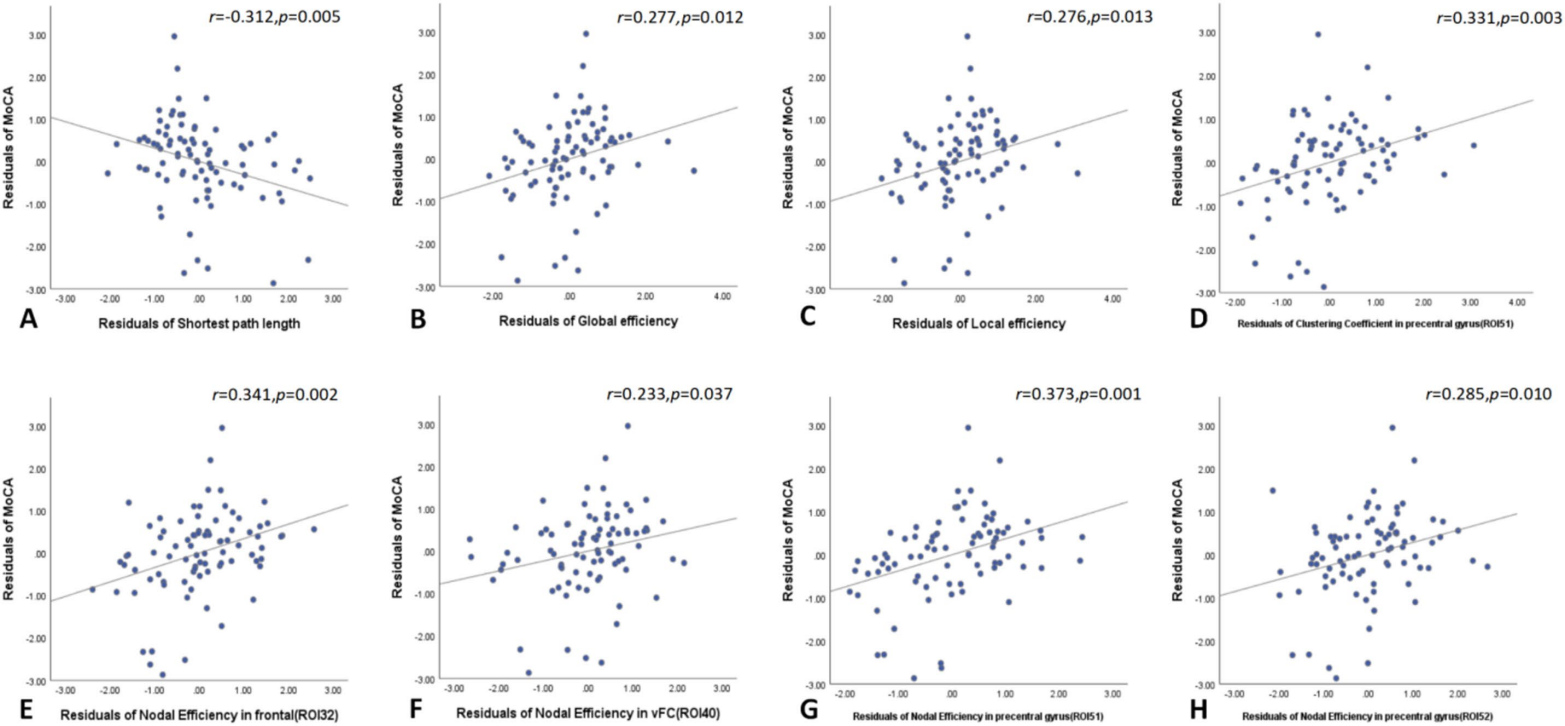
In the global network topology analysis, MoCA scores negatively correlated with shortest path length and positively correlated with both global and local efficiency in the T2DM group **(A–C)**. Node network topology analysis revealed that MoCA scores positively correlated with the clustering coefficient of the right precentral gyrus and with nodal efficiency in the right frontal cortex, left ventromedial frontal cortex, and both the right and left precentral gyri **(D–H)**. MoCA, Montreal cognitive assessment.

In the global network topology analysis, MoCA scores negatively correlated with shortest path length and positively correlated with both global and local efficiency in the T2DM group. Node network topology analysis revealed that MoCA scores positively correlated with the clustering coefficient of the right precentral gyrus and with nodal efficiency in the right frontal cortex, left ventromedial frontal cortex, and both the right and left precentral gyri (Figure 4).

### 3.6 Correlation between network parameters and laboratory indicators

**Figure 5:**
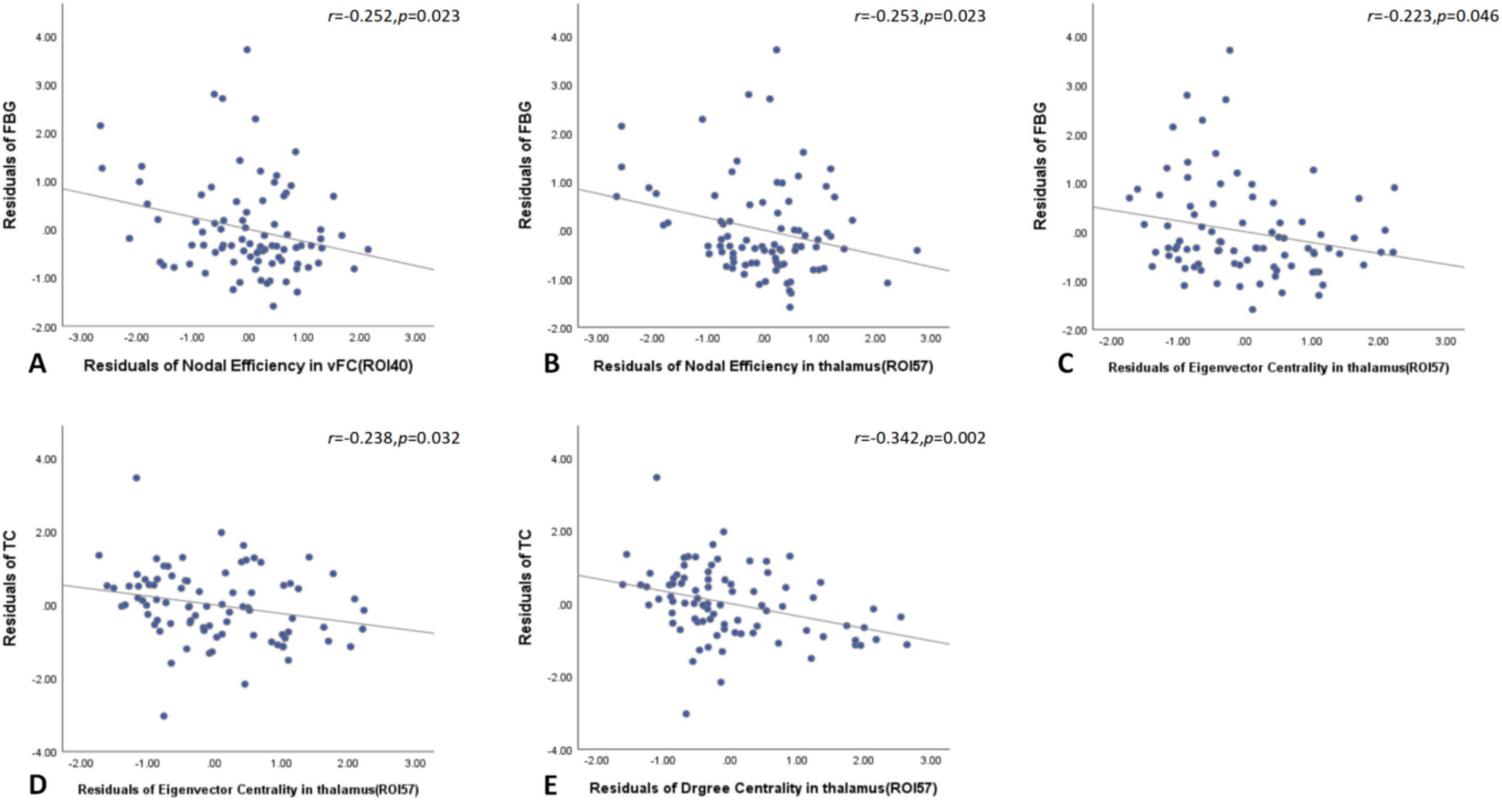
Node network topology analysis showed that FBG negatively correlated with nodal efficiency in the left ventromedial frontal cortex and left thalamus, as well as with eigenvector centrality in the left thalamus in T2DM patients **(A–C)**. Additionally, TG negatively correlated with both eigenvector and degree centrality in the left thalamus **(D,E)**. FBG, fasting blood glucose; TG, triglyceride.

Global network graph analysis revealed no significant correlations between network parameters and laboratory indicators in T2DM patients compared to the HC group. However, node network topology analysis showed that FBG negatively correlated with nodal efficiency in the left ventromedial frontal cortex and left thalamus, as well as with eigenvector centrality in the left thalamus. Additionally, TG negatively correlated with both eigenvector and degree centrality in the left thalamus (Figure 5).

## 4 Discussion

This study identifies significant alterations in brain functional connectivity in T2DM patients compared to healthy controls, suggesting that cognitive impairment in T2DM is influenced by interactions among different brain regions and networks.

The DMN plays a critical role in cognition-related functions such as memory, future planning, and social planning (Yeshurun et al., 2021). Consistent with previous findings, connectivity within the DMN network as well as connectivity between the DMN and other networks, particularly involving the posterior cingulate gyrus, prefrontal cortex, precuneus, and angular gyrus reduced in T2DM patients (Deng et al., 2021; Chen et al., 2016). Meng et al. demonstrated large-scale network damage centered on DMN in T2DM patients through a meta-analysis, which provides key insights into the neural mechanisms of diabetes-related cognitive decline (Meng et al., 2022). Significantly decreased connectivity between the DMN and the FPN was also observed (Meng et al., 2022), suggesting deficits in execution, attention and emotional regulation in T2DM patients (Nielsen et al., 2017). Previous report about increased connectivity in the pre-DMN and decreased connectivity in the post-DMN (Cui et al., 2015), both increased and decreased connections between DMN and SMN networks were also found in our study, dissociative patterns in the DMN likely triggered the T2DM-related cognition decline.

Additionally, our results reveal reduced functional connectivity between the SCN and several other networks, including the VN, DMN, and SMN. This suggests that diminished connectivity in T2DM affects both cognitive regions and integration centers. Significant connectivity reductions were also found in the three sensory networks—the VAN, SMN, and VN—correlating with abnormalities in attention, sensation, and vision reported in T2DM patients (Zhang et al., 2015; Xia et al., 2015; Faskowitz et al., 2022). The VN, in particular, is highly susceptible and often shows disruptions in T2DM (Ni et al., 2024; Kaewput et al., 2019). While T2DM is commonly associated with disconnections in the DAN (Meng et al., 2022), our study found only a slight abnormality in the connection between DAN and DMN. Possibly due to the relatively short duration of diabetes in our study cohort.

T2DM impairs brain functional network connectivity through several mechanisms. It leads to neuron loss, disrupts cell proliferation in the dentate gyrus, and damages synaptic plasticity, as evidenced by both human and animal studies (Jackson-Guilford et al., 2000; Li et al., 2002; Stranahan et al., 2008). Neuroplasticity, which involves structural and functional adjustments in response to stimuli or damage (Cramer et al., 2011; Fu and Zuo, 2011), is crucial here. Enhanced functional connectivity often reflects compensatory mechanisms that the nervous system employs to preserve normal function and optimize between-network interactions (Jing et al., 2023; Fang et al., 2019). Additionally, treatments such as blood glucose reduction and improved perfusion of small blood vessels can enhance network connectivity. For example, intranasal insulin has been found to improve resting-state functional connectivity in the hippocampus (Zhang et al., 2015), potentially explaining our observations of increased connectivity within the VN and between the DMN and the SMN. This is consistent with previous findings of increased connectivity between the thalamus and VN in pre-T2DM patients (Jing et al., 2023).

We compared 7 global indicators between the two groups. We believed that these 7 indicators were independent of each other, so we did not conduct multiple contrast correction for these 7 indicators, which is similar to most relevant studies (Shang et al., 2023; Xin et al., 2024). Whole-brain network analysis revealed that T2DM is associated with decreased global network connectivity, characterized by lower clustering coefficient, global efficiency, and local efficiency, as well as increased shortest path length (Farahani et al., 2019; van Bussel et al., 2016; Yang et al., 2020). The clustering coefficient, which measures the interconnection among adjacent nodes, decreased in T2DM, indicating reduced information processing within clustered brain regions. Our results also show diminished global and local efficiency, suggesting less effective network processing, and an increased shortest path length, reflecting slower information transmission between brain regions. Although some studies have reported increased clustering coefficient, global efficiency, and local efficiency with a decreased shortest path length in T2DM (van Bussel et al., 2016; Xin et al., 2024; Zhou et al., 2022; Zhang et al., 2021), our findings are consistent with observations in dementia, where functional connectivity is notably reduced and information processing efficiency is compromised (delEtoile and Adeli, 2017). The increased connectivity seen in early T2DM may be a compensatory response, yet it results in reduced between-network efficacy. These similar topological changes in T2DM and dementia suggest that T2DM may be a risk factor for developing dementia.

In nodal brain network analysis, T2DM is associated with a decreased clustering coefficients of the left basal ganglia and bilateral anterior central gyrus, indicating reduced connectivity between any one of three brain regions and adjacent brain regions, which would reduce the speed of information processing between adjacent node brain regions. The study of degree centrality helps to identify important nodes, and the reduced degree centrality in the bilateral thalamus and right basal ganglia suggests these areas are less synchronized in the network, resulting in a lower degree of integration of the entire brain network. Eigenvector centrality is a hierarchical measure as the sum of the centrality for any one node adjacent one and represents the “hub” in the functional brain network (Lorenzini et al., 2023). Decreased eigenvector centrality in the bilateral thalamus represents a weakened role as a “transit station” connecting to other essential brain regions. Overall, reduced nodal efficiency across multiple brain regions in T2DM signifies diminished information processing capability (Yang et al., 2020; delEtoile and Adeli, 2017; Jacob et al., 2022), supporting our study of a damaged state in T2DM patients. Some previous studies have concluded that the topology of T2DM networks with different states is mainly characterized by reduced efficiency (van Bussel et al., 2016; Xin et al., 2024). Our study results also support this view, indicating that the node network of T2DM patients included in our study is in a damaged state. The observed reduction in eigenvector centrality, an aspect infrequently highlighted in previous studies, indicates a notable alterations in network connectivity as a “hub” region in brain networks. Comprehensive brain and nodal graph theory analyses reveal a reduction in information processing efficiency in both nodes and connections across extensive brain networks.

Insulin, a growth factor with neurotrophic properties, is essential for regulating learning and memory (Rachdaoui, 2020). In T2DM, characterized by hyperinsulinemia, insulin resistance in the brain can arise from reduced insulin receptor expression and receptor-activating enzymes. This resistance can lead to the accumulation of amyloid *β*-protein (Aβ) and Tau proteins, contributing to cognitive decline (Arnold et al., 2018). The MoCA effectively detects mild cognitive impairment (Li et al., 2018), and lower MoCA scores in T2DM patients are linked to decreased efficiency in large-scale brain network connections. Poor glycemic control is associated with reduced global and local network efficiency and increased shortest path length, exacerbating cognitive decline. Node analysis reveals that elevated FBG further diminishes node performance. Specifically, decreased performance in the precentral gyrus (PreCG), which is crucial for verbal motor memory tasks (Sakurai et al., 2018), correlates with declining MoCA scores. This decline may impair speech motor functions, affecting MoCA outcomes. Previous studies have also connected MoCA scores in T2DM with PreCG volume and noted that increased centrality in PreCG is linked to abnormalities in brain tissue connections (Roy et al., 2020). Additionally, elevated TG levels are associated with reduced efficacy in the left thalamus. Elevated lipids not only affect the brain functional network in T2DM patients, but also further aggravate the decreased brain functional network caused by elevated blood glucose (Kassab et al., 2023; Yang et al., 2023). These findings highlight the necessity of controlling both blood glucose and lipid levels to reduce brain damage in T2DM patients.

This study has several limitations. First, being cross-sectional, it does not capture the effects of T2DM duration on brain connectivity. Future research should address this by incorporating longitudinal follow-up. Second, incomplete data on medication use limits our ability to control for their potential effects on brain functional networks.

In summary, our study identified abnormalities in large-scale brain networks in T2DM patients, suggesting that cognition decline is caused by large-scale DMN-centered networks instead of one single region. Graph theory analysis revealed reduced efficacy in T2DM brain topology, with cognitive decline linked to diminished efficiency in both global and node-specific networks. These findings suggest that brain functional connectivity and topology could serve as valuable imaging biomarkers for understanding the biological mechanisms underlying cognitive impairment in T2DM.

## Data Availability

The raw data supporting the conclusions of this article will be made available by the authors, without undue reservation.
