# Supplementary material for: Altered brain functional network connectivity and topology in type 2 diabetes mellitus
Source: Front Neurosci. 2025 Jan 28;19:1472010. doi: 10.3389/fnins.2025.1472010 (PMC11811103; doi:10.3389/fnins.2025.1472010)
Supplement: Supplementary file 1 [file Table_1.DOC]

# 9 Supplementary Material

Supplementary table 1. The name, network and MNI coordinates of the nodes.

| ROI | Brain region | MNI coordinates | | | Network |
| --- | --- | --- | --- | --- | --- |
|  |  | X | Y | Z |  |
| 1 | vmPFC | 6 | 64 | 3 | DMN |
| 2 | aPFC | 29 | 57 | 18 | FPN |
| 3 | aPFC | -29 | 57 | 10 | FPN |
| 4 | mPFC | 0 | 51 | 32 | DMN |
| 5 | aPFC | -25 | 51 | 27 | DMN |
| 6 | vmPFC | 9 | 51 | 16 | DMN |
| 7 | vmPFC | -6 | 50 | -1 | DMN |
| 8 | aPFC | 27 | 49 | 26 | VAN |
| 9 | vent aPFC | 42 | 48 | -3 | FPN |
| 10 | vent aPFC | -43 | 47 | 2 | FPN |
| 11 | vmPFC | -11 | 45 | 17 | DMN |
| 12 | vlPFC | 39 | 42 | 16 | FPN |
| 13 | vmPFC | 8 | 42 | -5 | DMN |
| 14 | ACC | 9 | 39 | 20 | DMN |
| 15 | vlPFC | 46 | 39 | -15 | DMN |
| 16 | dlPFC | 40 | 36 | 29 | FPN |
| 17 | sup frontal | 23 | 33 | 47 | DMN |
| 18 | vPFC | 34 | 32 | 7 | VAN |
| 19 | ACC | -2 | 30 | 27 | FPN |
| 20 | sup frontal | -16 | 29 | 54 | DMN |
| 21 | ACC | -1 | 28 | 40 | FPN |
| 22 | dlPFC | 46 | 28 | 31 | FPN |
| 23 | vPFC | -52 | 28 | 17 | FPN |
| 24 | dlPFC | -44 | 27 | 33 | FPN |
| 25 | vFC | 51 | 23 | 8 | DMN |
| 26 | ant insula | 38 | 21 | -1 | VAN |
| 27 | dACC | 9 | 20 | 34 | VAN |
| 28 | ant insula | -36 | 18 | 2 | VAN |
| 29 | dFC | 40 | 17 | 40 | FPN |
| 30 | basal ganglia | -6 | 17 | 34 | VAN |
| 31 | mFC | 0 | 15 | 45 | VAN |
| 32 | frontal | 58 | 11 | 14 | VAN |
| 33 | vFC | -46 | 10 | 14 | FPN |
| 34 | dFC | 44 | 8 | 34 | DAN |
| 35 | dFC | 60 | 8 | 34 | SMN |
| 36 | dFC | -42 | 7 | 36 | FPN |
| 37 | vFC | -55 | 7 | 23 | DAN |
| 38 | basal ganglia | -20 | 6 | 7 | SCN |
| 39 | basal ganglia | 14 | 6 | 7 | SCN |
| 40 | vFC | -48 | 6 | 1 | VAN |
| 41 | pre-SMA | 10 | 5 | 51 | VAN |
| 42 | vFC | 43 | 1 | 12 | VAN |
| 43 | SMA | 0 | -1 | 52 | SMN |
| 44 | mid insula | 37 | -2 | -3 | VAN |
| 45 | frontal | 53 | -3 | 32 | SMN |
| 46 | precentral gyrus | 58 | -3 | 17 | SMN |
| 47 | thalamus | -12 | -3 | 13 | SCN |
| 48 | mid insula | -42 | -3 | 11 | VAN |
| 49 | precentral gyrus | -44 | -6 | 49 | DAN |
| 50 | parietal | -26 | -8 | 54 | DAN |
| 51 | precentral gyrus | 46 | -8 | 24 | SMN |
| 52 | precentral gyrus | -54 | -9 | 23 | SMN |
| 53 | precentral gyrus | 44 | -11 | 38 | SMN |
| 54 | parietal | -47 | -12 | 36 | SMN |
| 55 | mid insula | 33 | -12 | 16 | SMN |
| 56 | mid insula | -36 | -12 | 15 | SMN |
| 57 | thalamus | -12 | -12 | 6 | SCN |
| 58 | thalamus | 11 | -12 | 6 | SCN |
| 59 | mid insula | 32 | -12 | 2 | SMN |
| 60 | temporal | 59 | -13 | 8 | SMN |
| 61 | mid insula | -30 | -14 | 1 | SMN |
| 62 | parietal | -38 | -15 | 59 | SMN |
| 63 | inf temporal | 52 | -15 | -13 | DMN |
| 64 | parietal | -47 | -18 | 50 | SMN |
| 65 | parietal | 46 | -20 | 45 | SMN |
| 66 | parietal | -55 | -22 | 38 | DAN |
| 67 | precentral gyrus | -54 | -22 | 22 | SMN |
| 68 | temporal | -54 | -22 | 9 | SMN |
| 69 | parietal | 41 | -23 | 55 | SMN |
| 70 | post insula | 42 | -24 | 17 | SMN |
| 71 | basal ganglia | 11 | -24 | 2 | SCN |
| 72 | inf temporal | -59 | -25 | -15 | DMN |
| 73 | post cingulate | 1 | -26 | 31 | DMN |
| 74 | parietal | 18 | -27 | 62 | SMN |
| 75 | parietal | -38 | -27 | 60 | SMN |
| 76 | post insula | -30 | -28 | 9 | SMN |
| 77 | parietal | -24 | -30 | 64 | SMN |
| 78 | temporal | 51 | -30 | 5 | SMN |
| 79 | post parietal | -41 | -31 | 48 | DAN |
| 80 | post cingulate | -4 | -31 | -4 | SCN |
| 81 | fusiform | 54 | -31 | -18 | FPN |
| 82 | temporal | -41 | -37 | 16 | SMN |
| 83 | temporal | -53 | -37 | 13 | SMN |
| 84 | fusiform | 28 | -37 | -15 | VN |
| 85 | precuneus | -3 | -38 | 45 | DMN |
| 86 | sup parietal | 34 | -39 | 65 | SMN |
| 87 | precuneus | 8 | -40 | 50 | DAN |
| 88 | IPL | -41 | -40 | 42 | DAN |
| 89 | parietal | 58 | -41 | 20 | VAN |
| 90 | post cingulate | -8 | -41 | 3 | DMN |
| 91 | inf temporal | -61 | -41 | -2 | DMN |
| 92 | occipital | -28 | -42 | -11 | VN |
| 93 | post cingulate | -5 | -43 | 25 | DMN |
| 94 | precuneus | 9 | -43 | 25 | DMN |
| 95 | temporal | 43 | -43 | 8 | VAN |
| 96 | IPL | 54 | -44 | 43 | FPN |
| 97 | parietal | -55 | -44 | 30 | VAN |
| 98 | post parietal | -35 | -46 | 48 | DAN |
| 99 | sup temporal | 42 | -46 | 21 | DMN |
| 100 | IPL | -48 | -47 | 49 | FPN |
| 101 | angular gyrus | -41 | -47 | 29 | DMN |
| 102 | temporal | -59 | -47 | 11 | SMN |
| 103 | IPL | -53 | -50 | 39 | DMN |
| 104 | precuneus | 5 | -50 | 33 | DMN |
| 105 | occipital | -18 | -50 | 1 | VN |
| 106 | IPL | 44 | -52 | 47 | FPN |
| 107 | post cingulate | -5 | -52 | 17 | DMN |
| 108 | post cingulate | 10 | -55 | 17 | DMN |
| 109 | precuneus | -6 | -56 | 29 | DMN |
| 110 | IPS | -32 | -58 | 46 | FPN |
| 111 | post cingulate | -11 | -58 | 17 | DMN |
| 112 | IPS | 32 | -59 | 41 | DAN |
| 113 | angular gyrus | 51 | -59 | 34 | DMN |
| 114 | occipital | -34 | -60 | -5 | VN |
| 115 | occipital | 36 | -60 | -8 | VN |
| 116 | temporal | 46 | -62 | 5 | VN |
| 117 | angular gyrus | -48 | -63 | 35 | DMN |
| 118 | TPJ | -52 | -63 | 15 | DAN |
| 119 | occipital | -44 | -63 | -7 | DAN |
| 120 | occipital | 19 | -66 | -1 | VN |
| 121 | precuneus | 11 | -68 | 42 | DMN |
| 122 | occipital | 17 | -68 | 20 | VN |
| 123 | IPS | -36 | -69 | 40 | DMN |
| 124 | occipital | 39 | -71 | 13 | VN |
| 125 | occipital | -9 | -72 | 41 | FPN |
| 126 | occipital | 45 | -72 | 29 | DMN |
| 127 | occipital | 29 | -73 | 29 | VN |
| 128 | occipital | -2 | -75 | 32 | FPN |
| 129 | occipital | -29 | -75 | 28 | DAN |
| 130 | occipital | -16 | -76 | 33 | VN |
| 131 | occipital | -42 | -76 | 26 | DAN |
| 132 | occipital | 9 | -76 | 14 | VN |
| 133 | occipital | 15 | -77 | 32 | VN |
| 134 | occipital | 20 | -78 | -2 | VN |
| 135 | post occipital | -5 | -80 | 9 | VN |
| 136 | post occipital | 29 | -81 | 14 | VN |
| 137 | post occipital | 33 | -81 | -2 | VN |
| 138 | post occipital | -37 | -83 | -2 | VN |
| 139 | post occipital | -29 | -88 | 8 | VN |
| 140 | post occipital | 13 | -91 | 2 | VN |
| 141 | post occipital | 27 | -91 | 2 | VN |
| 142 | post occipital | -4 | -94 | 12 | VN |
